# Evidence for the association of the *SLC22A4 *and *SLC22A5 *genes with Type 1 Diabetes: a case control study

**DOI:** 10.1186/1471-2350-7-54

**Published:** 2006-06-23

**Authors:** Jose Luis Santiago, Alfonso Martínez, Hermenegildo de la Calle, Miguel Fernández-Arquero, M Ángeles Figueredo, Emilio G de la Concha, Elena Urcelay

**Affiliations:** 1 Immunology Department, Hospital Universitario San Carlos, Madrid, Spain; 2 Endocrinology Department, Hospital Ramón y Cajal, Madrid, Spain

## Abstract

**Background:**

Type 1 diabetes (T1D) is a chronic, autoimmune and multifactorial disease characterized by abnormal metabolism of carbohydrate and fat. Diminished carnitine plasma levels have been previously reported in T1D patients and carnitine increases the sensitivity of the cells to insulin. Polymorphisms in the carnitine transporters, encoded by the *SLC22A4 *and *SLC22A5 *genes, have been involved in susceptibility to two other autoimmune diseases, rheumatoid arthritis and Crohn's disease. For these reasons, we investigated for the first time the association with T1D of six single nucleotide polymorphisms (SNPs) mapping to these candidate genes: slc2F2, slc2F11, T306I, L503F, OCTN2-promoter and OCTN2-intron.

**Methods:**

A case-control study was performed in the Spanish population with 295 T1D patients and 508 healthy control subjects. Maximum-likelihood haplotype frequencies were estimated by applying the Expectation-Maximization (EM) algorithm implemented by the Arlequin software.

**Results:**

When independently analyzed, one of the tested polymorphisms in the *SLC22A4 *gene at 1672 showed significant association with T1D in our Spanish cohort. The overall comparison of the inferred haplotypes was significantly different between patients and controls (χ^2 ^= 10.43; p = 0.034) with one of the haplotypes showing a protective effect for T1D (rs3792876/rs1050152/rs2631367/rs274559, CCGA: OR = 0.62 (0.41–0.93); p = 0.02).

**Conclusion:**

The haplotype distribution in the carnitine transporter locus seems to be significantly different between T1D patients and controls; however, additional studies in independent populations would allow to confirm the role of these genes in T1D risk.

## Background

Type 1 diabetes (T1D) is a multifactorial autoimmune T-cell-mediated disease resulting from selective destruction of the insulin producing β cells in the pancreatic islets, leading to an absolute insulin deficiency. The risk of developing T1D is determined by a complex interaction between multiple genetic and environmental factors. Although susceptibility to disease is strongly associated with alleles in the major histocompatibility complex (MHC) [1,2], there are more than 20 putative T1D susceptibility regions identified by linkage and association studies [3,4]. At present, several non-MHC susceptibility loci with modest genetic effects have been clearly defined. However, it is well known that many non-MHC loci predisposing to T1D remain as yet undefined [5].

Type 1 diabetes is a chronic degenerative disease, with altered metabolism characterized by hyperglycemia and ketoacidosis and T1D patients depend on exogenous insulin to sustain life. The role of the carnitine system in cell metabolism is mainly known in the mitochondria, where the interaction between fatty acid and glucose metabolism is fundamental for cell energy production [6,7]. However, carnitine not only contributes to the transport of activated long-chain fatty acids into mitochondria for β-oxidation, but it also increases the sensitivity of cells to insulin [8]. Decreased plasma carnitine levels have been reported in patients with type 2 diabetes [9-11] and some studies have investigated the carnitine status in T1D, finding similar results [12-15].

Adequate carnitine levels are required for normal lipid metabolism and are important for energy metabolism [16]. One important component of the carnitine system is the plasma membrane carnitine transporters, named organic cation transporters (OCTN1 and OCTN2) encoded by the *SLC22A4 *and *SLC22A5 *genes, respectively. Both genes map to the cytokine gene cluster on chromosome 5q31 and show 88% homology and 77% identity in their sequences. Despite OCTN1 and OCTN2 are considered as carnitine transporters, only OCTN2 is a high-affinity human carnitine transporter, while the carnitine transport activity of OCTN1 is very low [17,18]. In fact, a recent study has reported that the main substrate of this transporter is the ergothioneine, an intracellular antioxidant with metal ion affinity, which is transported one hundred times more efficiently than carnitine [18]. OCTN2 is widely expressed in many adult tissues, among them in pancreas, and it participates, at least in part, in proton/organic cation antiport at the renal apical plasma membrane level [19].

Recent reports performed associations of some polymorphisms within the *SLC22A4 *and *SLC22A5 *genes with two other autoimmune complex diseases (rheumatoid arthritis and Crohn's disease) [20,21]. The purpose of this study was to investigate the influence of the *SLC22A4 *and *SLC22A5 *genes in type 1 diabetes risk in the Spanish population. Six SNPs along these genes were considered good markers to map this region: slc2F2 (rs3792876) and slc2F11 (rs 2306772), which are SNPs in the *SLC22A4 *gene, were originally associated with RA susceptibility [20]. Other polymorphisms within the same linkage disequilibrium (LD) block (slc2F1) and slc2F2 showed lack of association with T1D [22]. We have studied two additional SNPs in this gene: T306I (rs272893) and L503F (rs1050152, SNP located in exon 9 of *SLC22A4*). The OCTN2-promoter (rs2631367) is a transversion (-207G>C) disrupting a heat shock element in the promoter region of the *SLC22A5 *gene and it has been described, together with L503F, as etiologic variant in Crohn disease [21]. Finally, we analyzed an intronic SNP in the *SLC22A5 *gene: the OCTN2-intron (rs274559) in order to define haplotypes within these genes.

## Methods

### Patients

We studied 295 unrelated Spanish white T1D patients (149 men and 146 women) diagnosed according to the criteria of the American Diabetes Association (ADA) and 508 healthy controls recruited among blood donors. Both groups ethnically matched from the Madrid area. The age at onset for the T1D patients range from 1 to 55 years old (median age at onset 15 years) and all subjects were insulin-dependent at the time to study. The protocol followed the principles expressed in the Declaration of Helsinki and it was approved by the Hospital Ethics Committee.

### SNP genotyping

SNPs slc2F2 (rs3792876), slc2F11 (rs2306772), T306I (rs272893), L503F (rs1050152) and OCTN2-intron (rs274559) were genotyped by TaqMan Assays on Demand under conditions recommended by manufacturer (Applied Biosystems), with identification numbers: C__3170428_10, C__3170458_1_, C__3170445_1_, C__3170459_10 and C__1173605_1, respectively. For the SLC22A5 promoter -207G>C (rs2631367), a TaqMan Assay by Design was performed.

### Statistical analysis

Differences in allele or genotype frequencies for each marker were calculated by Chi-square, or Fisher's exact test when necessary. Associations were estimated by the odds ratio (OR) with 95% confidence interval (CI). Statistical analysis used Epi Info v. 6.02 (CDC Atlanta USA).

Maximum-likelihood haplotype frequencies were estimated by applying the Expectation Maximization (EM) algorithm implemented by the Arlequin software [23], with number of iterations set at 5000 and initial conditions at 50, with an epsilon value of 10^-7^. This software yields the estimated frequency of each haplotype, but it does not output the expected haplotypes for each individual. The frequency data were transformed into absolute numbers multiplying the frequencies presented in Table 3 by the total number of haplotypes in each group (patients and controls). Then, these values were introduced into contingency tables to calculate Chi-square and p-values.

## Results

**Figure 1:**
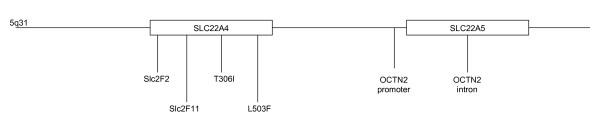
Schematic representation of the chromosomal region 5q31 with the relative position of the polymorphisms studied.

Six SNPs were studied in order to check the role of *SLC22A4 *and *SLC22A5 *genes in T1D predisposition (see figure 1). Slc2F2, Slc2F11, T306I and L503F map in the *SLC22A4 *gene and two additional SNPs, OCTN2-promoter and OCTN2-intron, are located in the *SLC22A5 *gene. The analysis of the control cohort showed complete linkage disequilibrium (LD) between Slc2F11 and slc2F2, and T306I was also found in complete LD with OCTN2-intron. Both SNPs (slc2F11 and T306I) were not considered in the subsequent case-control study, because they do not supply additional information. These polymorphisms conformed to Hardy-Weinberg equilibrium.

The association of the intronic slc2F2 polymorphism in the *SLC22A4 *gene with rheumatoid arthritis (RA) was originally reported in a Japanese population [20]. This SNP was even described as an etiological variant in Japan, being the mutant homozygous genotype (slc2F2*TT) associated with RA. However, this association could not be replicated either in British [24] or in Spanish populations [25]. Table 1 shows the phenotype and genotype distribution of this marker in the Spanish T1D patient and control cohorts, and lack of significant association with the disease was observed. The allelic distribution was similar to that previously observed in Spanish RA patients, (minor allele frequency slc2F2*T was 7.5% in RA and 9.5% in T1D patients). We tested the carrier rate of this allele (CT+TT vs. CC) to find a possible association with T1D, but a negative result was again obtained: OR = 1.30 (0.87–1.94); p = 0.17.

**Table 1:** Genotype and phenotype frequency distribution for OCTN1 markers.

Slc2F2	T1D (%)	Controls (%)
Alleles	2n = 590	2n = 1016
C	534 (90.5)	938 (92.3)
T	56 (9.5)	78 (7.7)
Genotypes	n = 295	n = 508
CC	240 (81.4)	432 (85)
CT	54 (18.3)	74 (14.6)
TT	1 (0.3)	2 (0.4)

L503F	T1D (%)	Controls (%)

Alleles	2n = 590	2n = 970
C	299 (50.7)	545 (56.2)
T	291 (49.3)	425 (43.8)
Genotypes	n = 295	n = 485
CC*	69 (23.4)	156 (32.2)
CT	161 (54.6)	233 (48.0)
TT	65 (22.0)	96 (19.8)

*OR = 0.64 (0.46–0.91); p = 0.009

Regarding the L503F (1672G>C) polymorphism, it was described by Peltekova et al. [21] as a functional variant associated with Crohn's disease, together with the OCTN2-promoter (-207G>C) variant. In fact, the authors found that the odds ratios conferred by the allele 1672*T, by the allele -207*C or by the TC minihaplotype were all similar. Both polymorphisms were also in strong linkage disequilibrium in our population (D' = 0.86) [26]. However, their etiological role could not be verified in Spanish Crohn's patients and they were not described as causative polymorphisms, but as genetic markers of risk or protection haplotypes. In our diabetic population, the allele 1672*T increased disease predisposition [OR = 1.25 (1.01–1.54); p = 0.034] and the 1672*CC genotype showed a protective effect (Table 1), but no significant differences between patients and controls were found for alleles and genotypes of the OCTN2-promoter (-207G>C) and of the other SLC22A5 intronic variant (Table 2).

**Table 2:** Distribution of OCTN2 polymorphisms.

OCTN2 Promoter	T1D (%)	Controls (%)
Alleles	2n = 590	2n = 1016
C	327 (55.4)	533 (52.5)
G	263 (44.6)	483 (47.5)
Genotypes	n = 295	n = 508
CC	91 (30.8)	150 (29.5)
CG	145 (49.2)	233 (45.9)
GG	59 (20.0)	125 (24.6)

OCTN2 Intron	T1D (%)	Controls (%)

Alleles	2n = 590	2n = 1016
A	370 (62.7)	636 (62.6)
G	220 (37.3)	380 (37.4)
Genotypes	n = 295	n = 508
AA	115 (39.0)	194 (38.2)
AG	142 (48.1)	248 (48.8)
GG	38 (12.9)	66 (13.0)

**Table 3:** The main haplotypes in region of OCTN1 and OCTN2 genes.

				T1D (2n = 590)		Controls (2n = 970)			
							
Slc2F2	L503F	OCTN2 Promoter	OCTN2 Intron	Frequency	Haplotype	Frequency	Haplotype	OR	p
C	T	C	A	0.47853	282	0.42690	414	1.23	0.05
C	C	G	G	0.27276	161	0.29604	287	0.89	0.33
C	C	G	A	0.06522	38	0.10018	97	0.62	0.02
C	C	C	A	0.07388	44	0.08832	86	0.83	0.33
T	C	G	G	0.09492	56	0.07732	75	1.25	0.22

Overall comparison between patients and controls: χ^2 ^= 10.43; p = 0.034

We continued our study by analyzing the inferred OCTN1-OCTN2 haplotypes, since haplotypes define better a DNA fragment where a susceptibility or protective gene could be located. Table 3 shows the haplotypes estimated by the Expectation-Maximization (EM) algorithm implemented by the Arlequin software with a frequency over 1%. The overall comparison of haplotypes in a 5 × 2 contingency table rendered a significant difference between patients and healthy controls (χ^2 ^= 10.43; p = 0.034), and being a unique omnibus comparison, this result does not need ulterior correction. These results evidenced one protection CCGA [OR = 0.62 (0.41–0.93); p = 0.02] and another risk CTCA [OR = 1.23 (1.00–1.52); p = 0.05] haplotypes, although they did not withstand correction for multiple testing (Table 3). The effect of the latter is secondary to the protective haplotype, as evidenced when this one is eliminated from the comparison and no significant predisposition effect was observed [OR = 1.16 (0.93–1.44); p = 0.18]; however, when the eliminated haplotipe is CTCA, a significant protection effect is still found for the CCGA haplotype [OR = 0.67 (0.44–1.02); p = 0.047). Moreover, as other haplotypes carrying the 1672*C allele do not show any influence in T1D predisposition, the protective effect of the 1672*C allele (p = 0.034) is due to this CCGA haplotype.

To complete the study in our population, we analyzed children and adolescents with type 1 diabetes fixing the cut-off age in 15 years old (median age at onset in our cohort). No differences between young patients and either adult patients or controls were found for any isolated variant or for the inferred haplotypes (data not shown). Finally, the frequencies of both, polymorphisms and inferred haplotypes, between male and female T1D patients were similar (data not shown).

## Discussion

The chromosomal region 5q31 contains several genes involved in immune and inflammatory responses and the *SLC22A4 *and *SLC22A5 *genes were associated with two autoimmune diseases (rheumatoid arthritis and Crohn's disease) [20,21]. Additionally, several genomewide scans for type 1 diabetes have identified susceptibility loci on different chromosomes, including the region 5q [27,28]. Moreover, carnitine has been described to increase sensitivity of cells to insulin [8], which could hypothetically lend cells more prone to an autoimmune attack. For all these reasons we considered that these genes could be candidates to modify the susceptibility to another autoimmune disease, as type 1 diabetes.

We tested six polymorphisms in the *SLC22A4 *and *SLC22A5 *genes and in five of them no significant independent association with type 1 diabetes could be found (Tables 1 and 2). The power of this study considering a relative risk of 1.5 was 75% for Slc2F2, 86% for L503F and 99% for OCTN2-promoter and OCTN2-intron, as calculated by an UCLA Department of Statistics sofware [29]. Therefore, we can exclude the studied *SLC22A5 *markers as causative candidates for T1D in Spanish patients, but in the case of Slc2F2 our results are not conclusive, albeit a recently published study does not find association of Slc2F1 and Slc2F2 *SLC22A4 *polymorphisms within a well-powered T1D cohort [22], in agreement with our data.

The overall distribution of the estimated haplotypes was significantly different between patients and healthy controls. Being ours the first study carried out in T1D, replication studies in different T1D populations would be necessary to firmly establish the role of these genes in autoimmune diabetes. We proved by using a stepwise procedure that the association with T1D of the protection haplotype CCGA is primary. The etiological polymorphisms previously found associated with increased susceptibility to RA (slc2F2*T) and to Crohn's disease (L503F*T) do not display a causative role by themselves in our population.

Low carnitine plasma levels have been found in children and adolescents with type 1 diabetes [12,13,15]. For this reason, we decided to conclude the study with an age-stratified analysis. No significant differences in the association of each polymorphism were observed when grouping by age at onset or gender. In the study of the inferred haplotypes again no differences were found between any group of patients and when we compared each group with controls. Therefore, it seems that the protective effect of the inferred haplotype is independent of both sex and age at disease onset.

A previous report about disequilibrium blocks at 5q31 in European-derived population [30] demonstrated limited haplotype diversity, concordantly with our data (5 haplotypes found out of the 2^4 ^= 16 theoretically possible). One of the defined blocks (92 kb long) corresponds to the carnitine transporter genes locus. These authors indicated that the chromosomal region 5q31 is divided into discrete blocks displaying complete linkage disequilibrium (LD). However, some degree of LD extending beyond the *SLC22A4 *and *SLC22A5 *genes could imply that most probably these genes, or other in LD with them, are responsible for the reported effect on T1D risk.

## Conclusion

In conclusion, the overall comparison of haplotypes within the chromosomal region where the carnitine transporter genes map seems to be different between type 1 diabetes patients and healthy controls in our Spanish population. The reduced carnitine plasma levels found in both type 1 and type 2 diabetes patients [12,13] could be explained by a mere increase in the activity of the carnitine transporters to supply the higher energetic cellular demand mainly provided by lipid metabolism in young diabetes patients. However, our results with the 1672*C allele and with the inferred haplotypes, the effect of carnitine sensitizing cells to insulin and potentially rendering them more amenable to an immune attack, and also the LD-block data described by Daly [30], support the role of the OCTN genes in T1D risk. Additional studies in independent populations and in both type 1 and type 2 diabetes patients will be needed to confirm the putative influence of the *SLC22A4 *and *SLC22A5 *genes in these diseases.

## Abbreviations

LD Linkage Disequilibrium

MHC Major Histocompatibility Complex

OCTN Organic Cation Transporter

SNP Single Nucleotide Polymorphism

SLC22 Solute Carrier Family 22

T1D Type 1 Diabetes

## Competing interests

The author(s) declare that they have no competing interests.

## Authors' contributions

JLS carried out the genotyping of the patients and a great part of the controls, participated in the statistical analysis and drafted the manuscript.

AM carried out a part of the genotyping of control samples and participated in the statistical analysis.

HdlC made the diagnosis and collaborated in collection of samples.

MAF participated in the genotyping and collection of samples.

MFA participated in the coordination of the study and helped to collect the DNA samples.

EgdlC coordinated the study and critically revised the manuscript.

EU conceived of the study, participated in the statistical analysis and wrote the major part of the manuscript.

All authors read and approve the final manuscript.

## Pre-publication history

The pre-publication history for this paper can be accessed here:


